# Targeting Ion Channels for Cancer Therapy: From Pathophysiological Mechanisms to Clinical Translation

**DOI:** 10.3390/ph18101521

**Published:** 2025-10-10

**Authors:** Sha Zhou, Xiong Song, Weian Zeng, Dongtai Chen

**Affiliations:** Department of Anesthesiology, State Key Laboratory of Oncology in South China, Collaborative Innovation Centre for Cancer Medicine, Guangdong Provincial Clinical Research Center for Cancer, Sun Yat-sen University Cancer Center, Guangzhou 510060, China; zhousha@sysucc.org.cn (S.Z.); songxiong@sysucc.org.cn (X.S.)

**Keywords:** ion channels, cancer, tumor biology, therapeutic target

## Abstract

Cancer remains a major global health burden, representing one of the leading causes of mortality among noncommunicable diseases worldwide. Although conventional treatment modalities, including surgical resection, chemotherapy, radiotherapy, targeted therapy, and immunotherapeutic interventions, have demonstrated clinical benefits, their therapeutic efficacy is often constrained by inherent limitations such as low specificity, systemic toxicity, or tumor heterogeneity. These challenges underscore the imperative for developing innovative treatment strategies. Emerging evidence has implicated ion channels as critical players in oncogenesis and cancer progression. These proteins modulate diverse oncogenic phenotypes, including uncontrolled proliferation, metastatic dissemination, and apoptotic resistance. Their frequent dysregulation in malignancies correlates with disease aggressiveness and clinical outcomes, positioning them as promising targets for precision oncology. Notably, pharmacological modulation of ion channels exerts multifaceted antitumor effects, with several channel-targeting agents advancing through clinical trials. This review explores recent advances in ion channel-targeted therapies, emphasizing their mechanisms, clinical applications, and challenges. Furthermore, we examine the pathophysiological contributions of ion channels to tumor biology and evaluate their emerging utility as predictive biomarkers, providing perspectives on addressing critical gaps in current oncologic management.

## 1. Introduction

Cancer remains one of the most formidable global public health challenges of the 21st century, with nearly 20 million new cases diagnosed annually and accounting for almost one-quarter of all deaths from noncommunicable diseases. Demographic projections indicate that the global cancer burden will escalate to approximately 35 million new cases per year by 2050 [1]. Despite significant progress in conventional antitumor strategies—including surgery, chemotherapy, radiotherapy, targeted therapy, and the rapidly evolving field of immunotherapy—used alone or in combination, a substantial proportion of patients continue to derive limited clinical benefit because the efficacy of these therapies is affected by factors such as low therapeutic specificity, severe toxicities, acquired resistance, limited applicable population, and dynamic tumor microenvironment [2,3,4,5]. As a result, clinical outcomes remain suboptimal for many patients, highlighting an urgent and unmet need for innovative therapeutic strategies.

The asymmetric distribution of ions across the cell membrane establishes electrochemical gradients that are fundamental to numerous cellular processes. Ion movement across the lipid bilayer is indispensable for maintaining cellular homeostasis and can be broadly classified into passive and active transport [6]. Active transport, exemplified by the sodium–potassium pump (Na^+^/K^+^-ATPase), requires direct ATP hydrolysis to move ions against their electrochemical gradient (from low to high concentration), thereby maintaining essential transmembrane gradients [7]. These gradients not only enable passive ion movement but also underpin a wide range of physiological functions. By contrast, passive transport relies on diffusion along the electrochemical gradient (from high to low concentration) without energy expenditure. It occurs either by simple diffusion across the lipid bilayer or via facilitated diffusion mediated by specific membrane transport proteins.

Ion channels are essential mediators of facilitated diffusion, enabling rapid and selective passive ion flux across cellular membranes in response to defined stimuli. This process is vital for maintaining the membrane potential (V_m_) and regulating a broad spectrum of physiological functions, including electrophysiological signaling, molecular transport, and cellular energy metabolism [8,9]. Based on their gating mechanisms, ion channels are commonly classified into four major categories [10]: (a) voltage-gated channels, which respond to changes in membrane potential to control cellular excitability; (b) ligand-gated channels, which undergo conformational changes upon ligand binding; (c) mechanosensitive channels, which are activated by mechanical forces such as pressure and contribute to processes like vascular tone and sensory transduction; and (d) thermosensitive channels, which are modulated by temperature fluctuations. Additionally, ion channels are categorized according to ion selectivity, most notably for sodium (Na^+^), potassium (K^+^), calcium (Ca^2+^), and chloride (Cl^−^) ions [11]. Dysregulation of these channels has been increasingly linked to tumorigenesis, influencing key processes such as cancer cell proliferation, migration, apoptosis, and resistance to therapy [12,13]. Aberrant expression patterns have also been associated with patient prognosis, highlighting ion channels as promising therapeutic targets in the context of precision oncology [14].

Targeting ion channels has emerged as a promising anticancer strategy, offering multifaceted therapeutic benefits, including the inhibition of tumor growth, enhancement of treatment sensitivity, and reversal of drug resistance (Table 1) [12]. Several ion channel-modulating agents have progressed into clinical evaluation, reflecting growing translational interest in this therapeutic avenue (Table 2) [15,16,17]. Given the breadth and complexity of research accumulated over the past three decades, this review does not aim to provide a comprehensive inventory of all ion channels implicated in tumorigenesis. Rather, it focuses on a selection of representative channels and their modulators, chosen for their established relevance to cancer hallmarks and the robustness of supporting preclinical and clinical evidence. Specifically, we examine their pathophysiological functions, current and potential clinical applications, and the principal challenges hindering their broader implementation. Due to constraints of length and scope, some ion channels, transporters, and cancer types of interest are not discussed.

## 2. The Role of Ion Channels in Cancer

### 2.1. Sodium Channel

Sodium ions (Na^+^) traverse the plasma membrane via multiple mechanisms, including the activity of the transmembrane Na^+^ gradient, Na^+^-dependent transporters, specific membrane channels, and energy-dependent processes [7]. Central to maintaining this critical gradient is the Na^+^/K^+^-ATPase, which hydrolyzes ATP to actively export Na^+^ and import K^+^. Sodium-selective channels—such as voltage-gated sodium channels (VGSCs), epithelial sodium channels (ENaCs), and acid-sensing ion channels (ASICs)—further mediate Na^+^ influx. This coordinated regulation results in an extracellular Na^+^ concentration approximately tenfold higher than that within the cytoplasm. Maintenance of Na^+^ homeostasis is indispensable for electrophysiological stability, and its disruption is a recognized pathogenic mechanism in acute inflammatory responses and ischemic injury [105,106]. Increasing evidence indicates that sodium imbalance also contributes to tumor progression, as malignant tissues often accumulate higher Na^+^ concentrations compared with normal tissues [107,108,109,110]. Such alterations may arise from aberrant Na^+^/K^+^-ATPase activity or dysregulated Na^+^ channel expression. Consequently, quantifying intratumoral Na^+^ concentration has been proposed to be a predictive biomarker for chemotherapy responsiveness [111].

VGSCs are integral membrane proteins that mediate Na^+^ flux across cellular membranes and are essential initiators of action potentials in excitable cells. They thereby govern electrical signaling and play fundamental roles in diverse physiological functions. Structurally, VGSCs are composed of a principal pore-forming α-subunit and one or more auxiliary β-subunits [112]. To date, nine mammalian α-subunit isoforms (Na_V_1.1–Na_V_1.9), encoded by SCN1A-SCN5A and SCN8A-SCN11A, have been identified. The β-subunits consist of five variants (β1a, β1b, β2, β3, and β4) encoded by *SCN1B*-*SCN4B*. Based on their sensitivity to tetrodotoxin (TTX), VGSCs are classified into two subtypes: TTX-resistant isoforms (Na_V_1.5, Na_V_1.8, and Na_V_1.9), which require micromolar TTX concentrations for inhibition, and TTX-sensitive isoforms, which are inhibited at nanomolar concentrations [113].

Aberrant VGSC expression and activity have been widely implicated in cancer biology. These channels promote tumor cell proliferation, invasion, and metastatic dissemination by modulating cellular excitability and downstream signaling. VGSCs are expressed across multiple malignancies, including colorectal, prostate, breast, lung, cervical, ovarian cancers, and melanoma. For example, Na_V_1.2 mRNA is upregulated in ovarian cancer [114], while Na_V_1.6 protein is markedly overexpressed in prostate and colorectal cancers [115,116]. In breast cancer, Na_V_1.5 is significantly overexpressed at both mRNA and protein levels compared with normal tissue, correlating strongly with disease recurrence and metastatic progression [117]. Mechanistic studies support these observations: siRNA-mediated silencing of Na_V_1.5 markedly inhibits proliferation and invasion of astrocytoma cells [118]. Na_V_1.6, upregulated in follicular thyroid carcinoma, promotes proliferation, epithelial–mesenchymal transition (EMT), and invasion through activation of the JAK2/STAT3 pathway [119]. Similarly, Na_V_1.7 drives gastric cancer progression via the MACC1-NHE1 axis [120]. In addition, the β1 subunit regulates cell–cell adhesion and migration in breast cancer [121].

Beyond VGSCs, ENaCs constitute another group of Na^+^ channels linked to oncogenesis. ENaCs, which mediate sodium reabsorption and water retention in renal collecting ducts, are fundamental to systemic fluid–electrolyte balance and blood pressure regulation. Their aberrant expression in malignancies such as hepatocellular carcinoma, melanoma, brain cancer, and breast cancer has been shown to facilitate tumor progression. ASICs, members of the ENaC/degenerin (ENaC/DEG) superfamily [122], also enhance oncogenic behaviors, including proliferation, EMT, and invasion, in colorectal and pancreatic cancers [123,124].

The tumor microenvironment (TME), comprising malignant cells, stromal elements, immune infiltrates, and the extracellular matrix (ECM), profoundly influences Na^+^ channel expression and activity. Hypoxia—a hallmark of solid tumors—induces Na^+^ channel upregulation, as demonstrated by increased Na_V_1.7 mRNA expression in prostate cancer cells [125]. In colorectal cancer, hypoxia-driven invasiveness depends on sustained Na^+^ current carried by the neonatal splice variant nNa_V_1.5 [126]. Moreover, extracellular acidosis in the TME modulates Na^+^ channel activity and exacerbates cancer progression. In breast cancer, a Na_V_1.5-dependent positive feedback loop enhances Na^+^ influx and glycolytic H^+^ export, thereby promoting extracellular acidification, ECM degradation, and tumor invasion [117].

### 2.2. Calcium Channel

As a vital second messenger, intracellular Ca^2+^ regulates fundamental biological processes, including proliferation, differentiation, migration, and programmed cell death [127,128]. Cells maintain an exceptionally low cytosolic Ca^2+^ concentration through active transport mechanisms, creating a steep 10- to 15,000-fold gradient relative to the extracellular milieu [128]. In response to diverse stimuli, transient elevations in cytosolic Ca^2+^ are initiated through release from the endoplasmic reticulum (ER) and Golgi apparatus (GA), efflux from mitochondria or lysosomes, and influx from the extracellular space. Termination of Ca^2+^ signaling occurs via sequestration into the ER, GA, or mitochondria, or by extrusion into the extracellular compartment.

**Figure 1:**
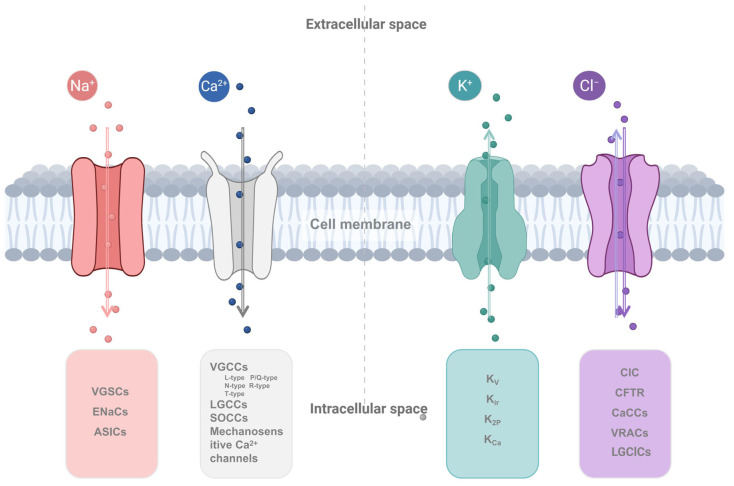
Classification and major subtypes of ion channels. Ion channels enable passive ion movement along electrochemical gradients and are categorized by their ion selectivity and gating mechanisms. The intensity of an ion current through a channel depends on its conductance and the electrochemical driving force, the difference between the membrane potential and the ion’s Nernst equilibrium potential. At rest, the driving force is strongly negative for sodium and calcium ions, so channel opening causes influx of these ions into the cell. For potassium, the driving force is positive, leading to efflux of these ions upon channel opening. Chloride ion flow direction through chloride channels may be inward or outward, depending on its transmembrane concentration gradient. These ion movements are critical for electrical signaling and cellular function.

Mounting evidence indicates that disruption of intracellular Ca^2+^ homeostasis accompanies malignant transformation, driving uncontrolled proliferation, enhanced survival, and metastatic potential. Ca^2+^ channels, which regulate cellular Ca^2+^ flux, are thus critically involved in tumor progression through alterations in their expression and function [129]. Plasma membrane Ca^2+^ channels are broadly divided into four classes: voltage-gated calcium channels (VGCCs), ligand-gated calcium channels (LGCCs), store-operated calcium channels (SOCCs), and mechanosensitive calcium channels (Figure 1). VGCCs open in response to membrane depolarization, and they share a conserved structure consisting of a pore-forming α1 subunit and auxiliary β subunits. Based on α1 subunit composition, they are classified into L-, P/Q-, N-, R-, and T-types, encoded by 10 distinct genes and grouped into three subfamilies (Ca_V_1.x, Ca_V_2.x, and Ca_V_3.x) [130]. LGCCs are activated by specific ligands and typically couple to intracellular signaling cascades. SOCCs are triggered by depletion of intracellular Ca^2+^ stores: when ER Ca^2+^ falls, the ER-resident protein stromal interaction molecule 1 (STIM1) undergoes conformational change, enabling interaction with the plasma membrane channel ORAI1. This opens ORAI1 to facilitate extracellular Ca^2+^ influx, replenishing ER stores via sarco/endoplasmic reticulum Ca^2+^-ATPase (SERCA)—a process termed store-operated Ca^2+^ entry (SOCE) [131]. Mechanosensitive Ca^2+^ channels, including Piezo and transient receptor potential (TRP) families, respond to mechanical stimuli such as stretch, compression, or membrane tension, mediating transmembrane Ca^2+^ influx [132].

Ca^2+^ channels function as pivotal regulators of intracellular Ca^2+^ influx, essential for sustaining physiological functions within neuronal networks, the cardiovascular system (including cardiac and smooth muscle), and skeletal muscle. Substantial evidence indicates that dysregulation of Ca^2+^ signaling is pathologically implicated in neurodegenerative disorders such as Parkinson’s disease; neurological conditions, including epilepsy; pain syndromes; autism spectrum disorder; and various cardiovascular dysfunctions [133]. Clinically, L-type Ca^2+^ channels are established pharmacological targets for widely used antihypertensive and antiarrhythmic agents, such as nifedipine and verapamil. The N-type Ca^2+^ channel is targeted by ziconotide for pain management [134,135]. Furthermore, gabapentinoids, which act on the α2δ-1 subunit of VGCCs, are employed in the treatment of neuropathic pain [136].

Recent research has underscored the critical role of Ca^2+^ channels in malignant tumors. Accumulating evidence indicates that Ca^2+^ signaling remodels TME and regulates key cancer cell behaviors, including proliferation, apoptosis, migration, and invasion. Specifically, He et al. demonstrated that Ca^2+^ influx promotes the polarization of tumor-associated macrophages (TAMs) from M2 toward M1 phenotype, thereby enhancing systemic antitumor immune responses [137]. Aberrant expression of multiple Ca^2+^ channels is a common feature in tumor cells. Notably, the VGCC subunit Ca_V_1.3 is frequently dysregulated in various cancers, with its encoding gene CACNA1D exhibiting elevated expression across diverse malignancies. In prostate cancer, CACNA1D mRNA and protein levels are significantly upregulated, and its overexpression correlates with an aggressive tumor phenotype [138]. Correspondingly, pharmacological blockade of Ca^2+^ channels has been associated with a reduced incidence of prostate cancer [139]. Similarly, Ca_V_1.3 dysregulation extends to breast cancer. Ca_V_1.3 is also highly expressed in breast cancer tissues. Downregulation of CACNA1D inhibits filopodia formation and suppresses the invasive capacity of breast cancer cells [140]. Beyond prostate and breast cancer, the α1D protein of Ca_V_1.3 is overexpressed in colorectal cancer and endometrial carcinoma compared to adjacent normal tissues. Silencing the *CACNA1D* gene (encoding α1D) disrupts Ca^2+^ homeostasis and impairs the migration of colorectal cancer and endometrial carcinoma cells [141,142]. Furthermore, dysregulation extends to other Ca^2+^ channels. The gene *CACNA1E*, encoding the α1 subunit of R-type VGCCs, is elevated in non-small-cell lung cancer (NSCLC) tissues and promotes NSCLC cell proliferation [143]. Amplification and overexpression of CACNA1E are also associated with disease relapse in favorable histology Wilms’ tumors [144].

SOCCs are also critically important in tumor biology. Yang et al. demonstrated that STIM1/ORAI1-mediated SOCE is indispensable for breast cancer cell migration and metastasis, establishing that Ca^2+^ influx via SOCE is a prerequisite for these processes. Genetic silencing of ORAI1 or STIM1 using RNA interference, as well as pharmacological blockade of SOCCs, significantly reduced metastatic burden in vivo [145]. Motiani et al. further identified ORAI3, a mammalian homolog of ORAI1, as a key contributor to the pathogenesis of estrogen receptor-positive (ER+) breast cancer [146,147,148]. Subsequent work by Faouzi et al. confirmed that ORAI3 regulates proliferation and cell-cycle progression in breast cancer cells [149,150]. In colorectal cancer (CRC), STIM1 overexpression correlates with tumor size, lymph node metastasis, depth of invasion, and elevated carcinoembryonic antigen levels. Mechanistic studies demonstrated that STIM1 enhances CRC cell migration by upregulating COX-2 expression and PGE_2_ production [151].

Mechanosensitive Ca^2+^ channels also play essential roles in tumor progression. Hasegawa et al. showed that the transcription factor YAP regulates Piezo1 expression in oral squamous cell carcinoma (OSCC), and that Piezo1 upregulation promotes OSCC cell proliferation [152]. Moreover, Piezo1 is highly expressed in human glioma and serves as an independent prognostic marker for patient outcomes [153].

TRP channels represent another major class of mechanosensitive Ca^2+^ channels. TRPs are nonselective cation channels that respond to diverse environmental and intracellular stimuli, and they are grouped into seven subfamilies based on sequence homology: TRPA (ankyrin), TRPC (canonical), TRPM (melastatin), TRPML (mucolipin), TRPN (nompC), TRPP (polycystin), and TRPV (vanilloid) [154,155]. With the exception of TRPN1, all subfamilies are expressed in mammals and fulfil critical physiological functions. In addition to their canonical roles in sensing thermal, mechanical, and chemical cues, TRP channels are increasingly recognized as regulators of cancer cell fate, modulating proliferation, apoptosis, and metastatic potential. Intriguingly, the identification of several TRP channels is historically linked to cancer research. For example, TRPM1 was initially discovered as a prognostic marker in melanoma, leading to its designation as “melastatin” [156].

TRPA1, the only member of the ankyrin subfamily, exhibits context-dependent effects in cancer. Under hypoxic conditions, TRPA1 suppresses invasion in lung cancer cells in vitro, whereas in breast and lung cancer spheroids, it enhances resistance to reactive oxygen species (ROS) and activates Ca^2+^-dependent anti-apoptotic signaling pathways [157]. Although TRPV1 is primarily localized to the plasma membrane and TRPV2 to intracellular membranes, both contribute to pro-survival signaling. Elevated TRPV1 expression has been reported in lung and breast cancers, where its overactivation promotes proliferation and migration of esophageal squamous cells [158,159]. TRPV2 expression is markedly increased in gastric cancer, where it has been proposed to be a prognostic biomarker, and in prostate cancer, where it is associated with castration resistance and metastatic progression [160,161].

Beyond their roles in survival signaling, TRP channels also regulate tumor angiogenesis and metastatic dissemination [162]. TRPV3 promotes angiogenesis in non-small-cell lung cancer (NSCLC) through the HIF-1α/VEGF pathway [163], while TRPV4 modulates mechanosensitivity in tumor endothelial cells to regulate vascular maturation [164]. TRPV5 and TRPV6, both highly Ca^2+^-selective channels with structural and functional homology, have also been implicated in cancer. TRPV6 is upregulated in thyroid, prostate, colon, and breast cancers [165], and its silencing suppresses pancreatic cancer progression [166]. Similarly, TRPC1 drives proliferation in follicular thyroid cancer via a Ca^2+^-dependent mechanism involving S1P3 and VEGFR2 modulation [167].

Among the TRPM subfamily, TRPM1 was the first identified in melanoma and is associated with tumor progression and survival, potentially through CaMKII-mediated activation of AKT signaling, thereby enhancing colony formation and tumor growth [168,169]. TRPM7 is the most extensively studied TRPM member in oncology. It has been shown to be indispensable for metastasis formation in breast cancer mouse models and is associated with poor prognosis in breast cancer patients [170]. Similarly, TRPM7 promotes migration in nasopharyngeal carcinoma (NPC) cells and correlates with unfavorable clinical outcomes [171]. TRPM8, initially identified as a cold- and menthol-sensitive receptor in prostate tissue [172], also exerts context-dependent functions in cancer. In breast cancer, TRPM8 activation inhibits proliferation and migration via AMPK signaling and autophagy modulation [173], whereas, in bladder cancer, it induces cell death through mitochondrial regulation [174].

Specific Ca^2+^ channels have also been linked to therapeutic resistance. TRPC6 has been shown to promote paclitaxel resistance in breast cancer cells by modulating integrin α6 mRNA splicing and TAZ activation. Pharmacological inhibition of TRPC6 with BI-749327 attenuates cancer stem cell-like properties and restores paclitaxel sensitivity in resistant organoids [30]. In gastric cancer, high TRPV2 expression correlates with advanced stage and poor prognosis, and mechanistically, TRPV2 confers cisplatin resistance by modulating cisplatin-induced apoptosis [175]. VGCC subunit Ca_V_1.3 contributes to resistance against androgen deprivation therapy in prostate cancer [176], while the α2δ-1 subunit encoded by *CACNA2D1* has been shown to enhance radioresistance in NSCLC [177].

Collectively, these findings demonstrate that Ca^2+^ channels regulate not only cancer initiation and progression but also therapeutic sensitivity. They therefore hold promise as diagnostic biomarkers and therapeutic targets in precision oncology.

### 2.3. Potassium Channel

Potassium ions (K^+^) represent the predominant intracellular cation in human cells, and their concentration is critical for maintaining cellular electrophysiological stability. K^+^ channels are the principal transmembrane conduits for K^+^ flux, regulating electrochemical gradients essential for action potential generation and cellular excitability.

Among ion channels, K^+^ channels display the greatest structural diversity and functional complexity. The Nomenclature Committee of the International Union of Basic and Clinical Pharmacology (NC-IUPHAR) has established a standardized classification system based on gating mechanisms and structural features [10]. Structurally, K^+^ channels are grouped into three major categories according to their number of transmembrane domains (TMs): 2TM, 4TM, and 6TM. Functionally, they are divided into four classes: voltage-gated potassium channels (K_V_), inwardly rectifying potassium channels (K_ir_), two-pore domain potassium channels (K_2P_), and calcium-activated potassium channels (K_Ca_) (Figure 1). K_V_ channels open or close in response to changes in membrane potential and are further subdivided into 12 subfamilies (K_V_1-12). K_ir_ channels exhibit strong inward rectification, characterized by significantly greater inward than outward K^+^ conductance. This property is crucial for maintaining the resting membrane potential and regulating cellular excitability. Based on structural and functional properties, K_ir_ channels are classified into seven subfamilies (K_ir_1-7). K_2P_ channels, also termed background or leak channels, provide basal K^+^ conductance and are divided into six subgroups: TWIK, TASK, TREK, TRESK, THIK, and TALK [178,179]. K_Ca_ channels are activated by intracellular Ca^2+^ and are subclassified according to their single-channel conductance: large conductance (BK_Ca_/MaxiK), intermediate conductance (IK_Ca_), and small conductance (SK_Ca_) [180].

Aberrant expression and dysregulation of K^+^ channels are observed across multiple cancer types, where they contribute to tumor initiation, progression, and clinical outcomes. In breast cancer, several K^+^ channels display dynamic expression changes. BK_Ca_ channels, for instance, show specific expression patterns that significantly correlate with clinical prognosis [181]. The oncogenic role of K^+^ channels is exemplified by the voltage-gated channel EAG1 (K_V_10.1), which is aberrantly expressed in diverse tumors and has been shown to promote cellular transformation in vitro [182,183,184]. KCNK1, a K_2P_ channel member, is highly expressed in breast cancer and promotes metabolic reprogramming through activation of lactate dehydrogenase A (LDHA) [185]. Similarly, elevated K_Ca_3.1 expression in breast cancer confers radioresistance by enhancing activation in response to ionizing radiation [186]. In lung cancer, the calcium-activated potassium channel KCNN4 is significantly overexpressed in lung adenocarcinoma, where it regulates apoptosis and EMT via the PI3K/AKT and MEK/ERK pathways [187]. TASK-1, a two-pore domain acid-sensitive K^+^ channel, modulates proliferation and apoptosis in non-small-cell lung cancer (NSCLC) [188]. Inwardly rectifying K^+^ channel K_ir_2.1 is upregulated in small-cell lung cancer (SCLC), with expression levels correlating with disease stage [189]. Beyond lung cancer, K_Ca_3.1 expression has prognostic significance in pancreatic ductal adenocarcinoma (PDAC) [190], while the voltage-gated K^+^ channel K_V_1.3 regulates apoptotic processes in melanoma [191].

### 2.4. Chloride Channel

As the most abundant anions in the human body, chloride ions (Cl^−^) are indispensable for maintaining osmotic pressure, cellular volume, and acid–base balance, as well as facilitating carbon dioxide transport through the chloride shift mechanism [192]. The transmembrane Cl^−^ gradient is tightly regulated by the coordinated action of passive Cl^−^ channels and active transporters.

Electrophysiological studies classify Cl^−^ channels into several major groups: the chloride channel (ClC) family, the cystic fibrosis transmembrane conductance regulator (CFTR), calcium-activated chloride channels (CaCCs), volume-regulated anion channels (VRACs), and ligand-gated chloride channels (LGClCs) [193]. The ClC family plays a central role in modulating membrane potential and maintaining Cl^−^ homeostasis within intracellular organelles. It consists of nine members (ClC-1 to ClC-7, ClC-Ka, and ClC-Kb), which exhibit diverse functions despite conserved sequence motifs. Evolutionarily, the ClC family has diverged into two functional classes: voltage-gated chloride channels and Cl^−^/H^+^ antiporters [192].

CFTR, a unique member of the ATP-binding cassette (ABC) transporter superfamily, is best known for the ΔF508 deletion mutation, which disrupts protein folding and trafficking, leading to cystic fibrosis (CF). Beyond its role in Cl^−^ conductance, CFTR also regulates the activity of other channels, including epithelial sodium channels (ENaCs) and CaCCs. CaCCs are predominantly formed by members of the TMEM16 family, with TMEM16A (ANO1) identified as the principal molecular determinant of CaCC function. VRACs facilitate regulatory volume decrease (RVD) by mediating Cl^−^ efflux in response to cell swelling [194]. These channels are heteromeric complexes that require LRRC8A as an essential structural component, which combines with other LRRC8 isoforms (LRRC8B-E) to form functional assemblies [195]. LGClCs, members of the Cys-loop ligand-gated ion channel superfamily, include γ-aminobutyric acid type A receptors (GABA_A-Rs) and glycine receptors (GlyRs), the predominant neuronal LGClCs mediating inhibitory neurotransmission in the central nervous system [196].

Dysregulation of specific Cl^−^ channels in cancer cells functionally contributes to survival and progression. As reviewed by Hong et al. [197], ClC-3 plays a particularly prominent role in cancer biology. In malignant glial cells, ClC-3 acts as a key regulator of the cell cycle [198]. In human glioma cells, ClC-3 is enriched at the plasma membrane and activated through Ca^2+^/calmodulin-dependent kinase II (CaMKII) signaling [199]. Genetic ablation of ClC-3 suppresses CaMKII-mediated chloride currents and reduces bradykinin-stimulated migration of glioma cells [200]. Similarly, silencing ClC-3 expression induces G0/G1 cell-cycle arrest and inhibits migration in nasopharyngeal carcinoma (NPC) cells [201,202]. In cervical carcinoma, both ClC-3 mRNA and protein are significantly upregulated compared with normal cervical tissues, and overexpression correlates with lymph node metastasis and poor prognosis [203].

While dysfunction of ClC channels has been implicated in diverse cancers, the role of CFTR in cancer risk is particularly notable in the context of cystic fibrosis (CF)—a multisystem genetic disorder caused by mutations in the CFTR gene. CF is associated with an increased risk of multiple malignancies through multifaceted mechanisms, including disruption of ion homeostasis, dysregulation of EMT and immune responses, and alterations in signaling pathways critical for proliferation and survival [204]. Clinical studies consistently support this association, particularly within the gastrointestinal tract. Initial reports in *The New England Journal of Medicine* identified a significantly increased risk of digestive tract cancers in CF patients [205], which was subsequently confirmed by long-term follow-up studies [206,207]. Notably, CF patients exhibit a 5-to-10-fold-higher risk of colorectal cancer (CRC), prompting recommendations for routine colonoscopic surveillance as the primary screening strategy for this population [208]. Elevated risks of pancreatic cancer, testicular cancer, and lymphoid leukemia have also been documented [207,209]. Nevertheless, the precise molecular mechanisms by which CFTR modulates tumor biology remain incompletely understood.

Beyond CFTR, other Cl^−^channels such as TMEM16A/ANO1 have been strongly linked to oncogenesis. TMEM16A encodes a member of the CaCC family, which regulates diverse physiological processes, including glandular secretion, smooth muscle contraction, and sensory transduction. Identified as a CaCC in 2008, TMEM16A has also been implicated in vascular regulation, emerging as a potential therapeutic target for hypertension [210,211,212,213,214]. Dysregulated ANO1 expression promotes oncogenic progression in multiple malignancies, including breast cancer, gastric cancer, and head and neck squamous cell carcinoma (HNSCC) [77,215,216]. Furthermore, aberrant ANO1 serves as a prognostic biomarker for CRC and pancreatic cancer [217,218]. Mechanistically, ANO1 activates ERK1/2 signaling and modulates the subcellular localization of p27Kip1, thereby regulating cell viability and apoptosis in HNSCC [215,219]. More recently, ANO1-mediated PI3K-AKT activation has been shown to impair ferroptosis in gastrointestinal cancers while simultaneously remodeling the tumor microenvironment through recruitment of cancer-associated fibroblasts (CAFs) and suppression of CD8^+^ T-cell activity [220].

Similarly, VRACs and LGClCs have been implicated in cancer biology. LRRC8A, the essential structural component of VRACs [194], has emerged as a promising prognostic biomarker across several malignancies [221,222]. For example, Zhang et al. demonstrated that LRRC8A promotes colon cancer metastasis by regulating the PIP5K1B/PIP2 signaling pathway [223]. With respect to LGClCs, growing evidence supports their involvement in tumorigenesis and progression. In breast cancer, the α3 subunit-encoding gene *GABRA3* is significantly upregulated in both clinical specimens and cell models. Interestingly, its RNA-edited isoform exerts tumor-suppressive effects by inhibiting cellular migration, invasion, and metastasis [224]. Conversely, the pi subunit-encoding gene *GABRP* promotes migration in basal-like breast cancer cells through ERK1/2 pathway activation [225].

### 2.5. Trace Metal Ion Channel

Unlike the major ions that typically diffuse passively through selective channels, trace elements predominantly rely on active transport mechanisms mediated by specialized transmembrane transporters. Distinct metalloprotein families exemplify this regulation: iron homeostasis is maintained by divalent metal transporter 1 (DMT1) and ferroportin (FPN); zinc balance is achieved through the coordinated actions of Zrt-/Irt-like protein (ZIP, SLC39A) importers and zinc transporter (ZnT) exporters; and copper transport is governed by copper transporter 1 (CTR1) and the P-type ATPases ATP7A/B. In addition to these dedicated transporters, increasing evidence highlights the contribution of nonselective cation channels, such as TRPM7 and TRPML1, in mediating the transmembrane flux of essential divalent cations (e.g., Zn^2+^, Mn^2+^, and Fe^2+^). These channels operate in concert with ATP-dependent transporters to ensure cellular metal homeostasis [226,227].

TRPM7, a unique bifunctional protein combining ion channel and kinase activities, exerts pleiotropic effects on tumor initiation, progression, and metastasis. Aberrant overexpression of TRPM7 has been reported across multiple cancers, where it regulates proliferation, migration, invasion, and EMT through both its ion-conducting and kinase functions [228]. For example, in pancreatic ductal adenocarcinoma PDAC, TRPM7 overexpression correlates with poor prognosis, and its kinase domain is required for interaction with PAK1 to maintain the mesenchymal phenotype of PDAC cells [229]. In breast cancer, TRPM7’s oncogenic role is partly mediated by Zn^2+^-dependent regulation of MDMX stability [230]. Beyond intrinsic tumor cell regulation, TRPM7 also shapes the TME: it mediates Mg^2+^ influx into macrophages, driving M2 polarization and thereby accelerating tumor progression [231].

Among the mucolipin TRP subfamily, TRPML1 (MCOLN1) is widely expressed and primarily localized to lysosomes. Lysosomal patch-clamp studies have confirmed TRPML1 as a nonselective cation channel facilitating efflux of divalent cations (Ca^2+^, Zn^2+^, Fe^2+^) from the lysosomal lumen. Recent findings implicate TRPML1 in regulating lysosomal functions that confer tumor resistance to ferroptosis and modulate autophagy [232,233]. Notably, TRPML1-mediated lysosomal exocytosis has been identified as a critical anti-ferroptotic mechanism. In AKT-hyperactivated cancers, TRPML1 is phosphorylated by AKT at Ser343, preventing ubiquitination-mediated degradation. Stabilized TRPML1 interacts with ARL8B to initiate lysosomal exocytosis, thereby reducing intracellular Fe^2+^ levels, enhancing membrane repair, and promoting resistance to ferroptosis. Importantly, a synthetic peptide targeting TRPML1 disrupts the TRPML1-ARL8B interaction, suppresses lysosomal exocytosis, amplifies ferroptosis, and enhances sensitivity of AKT-driven tumors to radiotherapy and immunotherapy in vivo [233]. Furthermore, TRPML1 has been reported to regulate oncogenic autophagy through Zn^2+^ influx, further underscoring its multifaceted role in cancer [234].

### 2.6. Ion Transporters

Similar to ion channels, ion transporters are essential membrane proteins that mediate ion movement across the lipid bilayer [235]. However, their operational mechanisms are fundamentally distinct: ion channels permit passive diffusion along electrochemical gradients, whereas ion transporters actively translocate ions against these gradients, typically through energy expenditure. Based on their functional mechanisms, ion transporters can be broadly divided into two categories: ion pumps and ion exchangers.

Ion pumps are active transporters that directly utilize ATP hydrolysis to move ions against their gradients, thereby establishing and maintaining transmembrane ion homeostasis. Classic examples include the sodium–potassium pump (Na^+^/K^+^-ATPase), which expels three Na^+^ and imports two K^+^ ions per ATP hydrolyzed to sustain resting membrane potential and cellular volume stability, and the calcium pump (Ca^2+^-ATPase), which extrudes Ca^2+^ to the extracellular space or sequesters it into the endoplasmic reticulum, maintaining low cytosolic Ca^2+^ essential for signaling. Proton pumps (H^+^-ATPases) are indispensable for gastric acid secretion and the acidification of intracellular organelles. By contrast, ion exchangers mediate secondary active transport, harnessing the energy stored in pre-established ion gradients rather than directly consuming ATP. For example, the Na^+^/Ca^2+^ exchanger (NCX) couples Ca^2+^ efflux to Na^+^ influx, while the Na^+^/H^+^ exchanger (NHE) regulates intracellular pH by extruding protons in exchange for Na^+^ uptake. Similarly, the Na^+^/K^+^/2Cl^−^ cotransporter (NKCC) facilitates the coupled transport of Na^+^, K^+^, and Cl^−^, thereby contributing to cell volume regulation and intracellular chloride homeostasis.

Ion transporters play fundamental roles in intracellular ion balance, pH regulation, volume control, and signal transduction. Dysregulation of their expression or activity has been widely implicated in malignancies. For instance, Na^+^/K^+^-ATPase is upregulated in gastric and breast cancers, where enhanced pump activity promotes invasion, tumor progression, and poor prognosis through activation of oncogenic PI3K/AKT signaling [236,237]. Ca^2+^-ATPases comprise three subtypes based on subcellular localization: plasma membrane Ca^2+^-ATPases (PMCAs), ER/sarcoplasmic reticulum Ca^2+^-ATPases (SERCAs), and Golgi/secretory pathway Ca^2+^-ATPases (SPCAs). PMCA2 is markedly overexpressed in certain breast cancer cell lines [238], whereas downregulation of PMCA4 and PMCA1 has been reported in colon and oral squamous cell carcinoma, respectively, resulting in elevated cytosolic Ca^2+^ and enhanced proliferation [239,240]. Among SERCAs, isoform-specific dysregulation has been associated with cancer [241]: SERCA2 is overexpressed in colorectal cancer and promotes proliferation and migration [242], while SERCA3 expression declines during colon tumor progression despite its initial elevation during differentiation [243]. In breast cancer, SPCA1 is preferentially expressed in basal-like subtypes but reduced in luminal tumors [244]. SPCA2 is upregulated in breast cancers, where knockdown suppresses proliferation and xenograft tumor growth, while overexpression enhances proliferation and activates ERK1/2 signaling through constitutive Ca^2+^ entry [245].

Although studies on NCX in cancer remain limited, elevated NCX3 transcript levels have been observed in therapy-resistant ovarian carcinoma and medulloblastoma cells compared with sensitive counterparts [88,89,246]. NHE1, the predominant isoform mediating proton efflux, is consistently upregulated in diverse malignancies. Hyperactive NHE1 promotes malignant progression by maintaining intracellular alkalinization, generating an acidic tumor microenvironment, regulating cell volume, altering morphology, and enhancing migration. Elevated NHE1 expression has been documented in gliomas [247], and it contributes to 5-fluorouracil resistance in gastric cancer cells via JAK/STAT3 signaling [248]. NKCC1 is similarly dysregulated in cancer, with overexpression enhancing proliferation and invasion in HCC [249]. In gastric cancer, NKCC1 promotes malignant progression by activating the MAPK–JNK pathway and inducing EMT [250].

## 3. Ion Channels as Pharmacological Targets in Cancer

The recognition of ion channels as critical regulators of oncogenesis has stimulated extensive research into their therapeutic potential. Two key questions now dominate the field: Is targeting ion channels a viable strategy to mechanistically disrupt oncogenic processes? And do these targets hold genuine promise for successful clinical translation into novel therapeutics?

### 3.1. Na^+^ Channel Inhibitors

Several pharmacological classes—including local anesthetics, antiarrhythmic agents, and antiepileptic drugs—exert their therapeutic effects primarily through inhibition of Na^+^ channels. Preclinical studies have shown that agents such as lidocaine, ropivacaine, phenytoin, and carbamazepine significantly modulate cancer cell behavior. Lidocaine, one of the most widely used local anesthetics in clinical practice, primarily functions by blocking VGSCs [251]. Recent studies demonstrate that lidocaine suppresses ovarian cancer metastasis via inhibition of Na_V_1.5-mediated EMT and reduces pulmonary metastasis in a murine breast cancer surgical model [18,20]. Similarly, phenytoin has been shown to decrease migration and invasion in metastatic breast cancer by selectively targeting Na_V_1.5 [21]. Ropivacaine enhances sorafenib-induced apoptosis in HCC cells through suppression of the IL-6/STAT3 signaling pathway [22].

In addition to these conventional drugs, peptide toxins and monoclonal antibodies targeting specific VGSC isoforms represent innovative strategies in oncology. For example, the spider venom-derived toxin JZTX-14 selectively blocks Na_V_1.5, thereby inhibiting breast cancer cell proliferation and migration [23]. Likewise, the peptide toxin HNTX-III, which targets Na_V_1.7, regulates migration and invasion in rat prostate cancer cells [25]. Furthermore, a polyclonal antibody against the neonatal splice variant of Na_V_1.5 significantly reduces the invasive capacity of MDA-MB-231 breast cancer cells in a dose-dependent manner, underscoring its therapeutic potential in breast cancer [24].

Considerable attention has also been directed toward the synergistic potential of Na^+^ channel modulators with established anticancer therapies. For instance, Sui et al. reported that the Na_V_1.5 activator veratridine increased the chemosensitivity of colorectal cancer cells to 5-fluorouracil [252]. Lidocaine enhances the sensitivity of ovarian cancer cells to carboplatin and paclitaxel [18,253] and restores temozolomide (TMZ) responsiveness in glioblastoma by inhibiting the HGF/MET pathway [19]. In the context of radiotherapy, the use of antiepileptic drugs has been associated with improved prognosis in breast cancer patients with brain metastases undergoing whole-brain radiotherapy [254].

The impact of Na^+^ channel inhibitors on antitumor immunity remains incompletely understood; however, emerging evidence suggests that certain local anesthetics may exert immunomodulatory effects. Preclinical studies have demonstrated that lidocaine and ropivacaine possess significant immune-regulatory properties. In vitro experiments using clinically relevant concentrations of lidocaine revealed enhanced natural killer (NK) cell activity [255]. Additionally, ropivacaine has been shown to upregulate MHC-I expression on tumor cells, thereby facilitating recognition and elimination by cytotoxic T lymphocytes within the TME [256].

Clinical investigations evaluating Na^+^ channel modulators are currently underway in various malignancies. An open-label, multicenter, randomized trial reported that peritumoral lidocaine injection prior to surgery significantly improved both disease-free survival (DFS) and overall survival (OS) in patients with early breast cancer (NCT01916317) [95]. In colorectal cancer, a prospective study of 150 surgical patients demonstrated that those receiving sevoflurane anesthesia combined with a 48 h lidocaine infusion exhibited a significantly lower one-year recurrence rate compared with patients receiving sevoflurane alone (NCT02786329) [15]. Furthermore, riluzole, another VGSC inhibitor, demonstrated favorable tolerability in a phase I trial investigating its combination with mFOLFOX6 and bevacizumab in metastatic colorectal cancer patients (NCT04761614) [96].

### 3.2. Ca^2+^ Channel Inhibitors

Calcium channel blockers (CCBs), a class of agents that inhibit Ca^2+^ influx through voltage-gated or other calcium channels, are widely used in the management of cardiovascular dysfunction, psychiatric disorders, and neurodegenerative diseases. In recent years, their potential applications in oncology have attracted increasing attention.

Commercially available antitumor agents targeting Ca^2+^ channels have primarily focused on VGCCs. Several FDA-approved CCBs, including lacidipine, manidipine, lomerizine, and benidipine, have demonstrated efficacy in inhibiting stem cell-like properties and inducing apoptosis in ovarian CSCs [257]. Bepridil, commonly prescribed for arrhythmia and heart failure, suppresses EMT-associated gene expression in ovarian cancer and exhibits antitumor activity in chronic lymphocytic leukemia through attenuation of NOTCH1 activation [26,27]. Diltiazem has been shown to modulate EMT and inhibit metastasis in triple-negative breast cancer (TNBC) mouse models [28]. In addition, the T-type CCB KTt-45 exerts anticancer effects by inducing mitochondrial-dependent apoptosis in HeLa cells [29].

Blockade of TRP channels also demonstrates substantial therapeutic promise. For example, inhibition of TRPC6 by BI-749327 significantly reduces mammosphere formation in breast CSCs [30]. Both natural compounds and synthetic analogues targeting TRP channels have exhibited anticancer properties across diverse tumor types. Cannabinoids, acting as TRP channel blockers, induce tumor cell death, inhibit angiogenesis and invasion, and modulate antitumor immunity [258]. In orthotopic HCC xenograft models, cannabinoids inhibited tumor growth through AMPK activation and regulation of autophagy [31]. In lung cancer, cannabinoids reduced invasion by modulating ICAM-1 and TIMP-1 expression [32]. However, it is noteworthy that delta-9-tetrahydrocannabinol has been reported to promote breast cancer growth and metastasis by impairing antitumor immunity, mediated via CB2 receptor-driven Th1/Th2 imbalance [259]. Monoclonal antibodies targeting Ca^2+^ channels represent another emerging therapeutic approach. For instance, mAb82, directed against TRPV6, induces apoptosis and suppresses tumor growth in prostate cancer models [33].

Moreover, specific CCBs have demonstrated efficacy in enhancing sensitivity to, or reversing resistance against, anticancer therapies. A recent study reported that the L-type CCB lacidipine potentiates the effects of doxorubicin and cisplatin in TNBC by selectively targeting Ca^2+^ channels to inhibit the Pyk2-JAK1–calmodulin complex-mediated IDO1 immunosuppressive pathway [34]. Similarly, lercanidipine and amlodipine inhibit the YY1/ERK/TGF-β axis, significantly sensitizing gastric cancer cells to doxorubicin [35]. The T-type CCB mibefradil (MIB) synergizes with carboplatin, promoting apoptosis and restoring platinum sensitivity in resistant ovarian tumors in murine xenograft models [36]. In gliomas, MIB induces glioblastoma cell apoptosis while suppressing glioblastoma stem-like cell phenotypes, thereby enhancing chemosensitivity to temozolomide [37,260]. Pharmacological inhibition of SOCE with SKF96365 augments 5-FU chemosensitivity in HCC through autophagic mechanisms [38]. In small-cell lung cancer patient-derived xenograft (PDX) models, the α2δ1 subunit-specific monoclonal antibody 1B50-1 enhanced the efficacy of chemotherapy and delayed relapse, highlighting its therapeutic potential in both combination and sequential regimens [39]. TRPV1 has also been implicated in chemoresistance: it promotes cisplatin resistance in cervical cancer via the Ca^2+^–AMPK pathway, whereas pharmacological inhibition with the antagonist AMG9810 reverses cisplatin resistance [40]. Similarly, pharmacological inhibition of TRPM2 with compound D9 synergizes with osimertinib, a third-generation EGFR-TKI, effectively inducing apoptosis and reducing viability in osimertinib-resistant lung cancer cells [41]. In addition to chemosensitization, CCBs also augment antitumor immune responses. Wu et al. demonstrated that nifedipine suppresses colorectal cancer immune evasion via NFAT2-dependent mechanisms and further enhances the efficacy of PD-1 blockade in preclinical murine models [42].

Encouraged by robust preclinical evidence, several clinical trials have been initiated to evaluate CCBs as novel oncological agents. A phase I trial (NCT01480050) assessed the safety and tolerability of MIB administered sequentially with TMZ in patients with recurrent high-grade gliomas (rHGGs). At the maximum tolerated dose (MTD), this regimen was well tolerated, demonstrating a favorable safety profile [16]. Another phase I trial (NCT02202993) conducted at Yale University investigated the combination of mibefradil dihydrochloride with concurrent hypofractionated radiotherapy in progressive or recurrent glioblastoma, although results have not yet been published. An open-label, dose-escalation phase I trial (NCT01578564) evaluating the TRPV6 inhibitor SOR-C13 demonstrated acceptable safety and tolerability in patients with advanced epithelial-derived solid tumors [97]. The ARISTOCRAT trial (NCT05629702), a multicenter, phase II, double-blind, placebo-controlled randomized study, is currently assessing the efficacy of cannabinoids combined with TMZ in recurrent glioblastoma [98]. Furthermore, a monoclonal antibody targeting a variant of P2X7 (nfP2X7) completed phase I trials in basal cell carcinoma (NCT02587819), demonstrating promising efficacy, with 65% of participants exhibiting measurable reductions in lesion area following 28 days of daily administration [99].

Carboxyamidotriazole (CAI), an inhibitor of non-voltage-gated Ca^2+^ channels and Ca^2+^-mediated signaling pathways, has also demonstrated potent anticancer activity by suppressing angiogenesis, tumor growth, and metastasis. Although CAI promotes IFN-γ secretion by T cells, it paradoxically enhances immune evasion through the IDO1-Kyn-AhR axis. Notably, combining CAI with IDO1 or AhR antagonists reduces PD-1 expression, improves CD8^+^ T-cell infiltration and effector function, suppresses tumor growth, and prolongs survival in murine models [261]. Several clinical trials have evaluated CAI’s safety and efficacy [17,100,104]. A phase II trial in relapsed epithelial ovarian cancer indicated its potential for disease stabilization [104], while a phase III randomized controlled trial in advanced NSCLC (CTR20160395) demonstrated that CAI combined with platinum-based chemotherapy improved progression-free survival, supporting its therapeutic potential [101].

### 3.3. K^+^ Channel Inhibitors

K^+^ channels represent promising therapeutic targets in oncology. Several pharmacological agents targeting distinct K^+^ channel subfamilies, including voltage-gated (K_V_), calcium-activated (K_Ca_), two-pore domain (K_2P_), and inwardly rectifying (K_ir_) channels, have demonstrated antitumor activity in both in vitro and in vivo models.

Among K_V_ channels, K_V_1.3, K_V_10.1, and K_V_11.1 have emerged as prominent therapeutic candidates. FS48 from *Xenopsylla cheopis* salivary glands inhibits migration and invasion of human lung cancer cells by blocking K_V_1.3 [43]. KAaH1 from scorpion venom suppresses migration and adhesion of glioblastoma cells by targeting K_V_1.1 and K_V_1.3 [44]. Psoralen derivatives induce tumor cell death by inhibiting mitochondrial K_V_1.3 expression and inducing mitochondrial dysfunction [45]. Memantine triggers acute lymphoblastic leukemia (ALL) cell death via K_V_1.3 inhibition, mechanistically linked to suppression of AKT and ERK1/2 signaling [47]. Small-molecule inhibitors such as Psora-4, PAP-1, and clofazimine reduce proliferation and induce apoptosis via Kv1.3 channel blockade [262]. Two PAP-1 derivatives, PAPTP and PCARBTP, enhance the efficacy of cisplatin in murine melanoma models [45]. Other Kv1.3 inhibitors, including the plant-derived polycyclic compound genistein, exert antiproliferative and pro-apoptotic effects in cancer cells [48,49]. K_V_10.1 has also been extensively investigated as an oncogenic driver. Multiple agents, including Chloroquine, Astemizole, Procyanidin B1, Tetrandrine, and Nutlin-3, selectively target K_V_10.1 to suppress malignant proliferation and migration [50,51,52,53,54]. Similarly, agents such as celastrol, berberine, resveratrol, and clarithromycin modulate cancer cell fate via K_V_11.1-dependent mechanisms [55,56,57,58].

Targeting K_Ca_ channels is also an emerging anticancer strategy. The BK_Ca_ blocker Paxilline prevents hypoxia-induced migration in glioblastoma cells [59] and, together with Iberiotoxin, regulates cell cycle and proliferation in neuroblastoma cells [60]. Iberiotoxin impairs proliferation and migration in endometrial cancer cells [61] and inhibits remote colonization of HCC cells in xenograft models [62]. Tetraethylammonium (nonselective BK_Ca_ blocker) induces G2 phase arrest and reduces viability of HCC cells under hypoxic conditions, while Penitrem A (selective BK_Ca_ blocker) induces G1 phase arrest and exerts antiproliferative effects in breast cancer [62,63]. Trimebutine maleate downregulates stemness-related protein expression and slows the growth of ovarian CSCs [64]. Pharmacological or genetic blockade of IK_Ca_ and SK_Ca_ channels effectively curbs tumor progression. Temozolomide and Vigabatrin decrease IK_Ca_ current amplitude in glioma cells, potentially contributing to their antineoplastic effects [65,66]. Clotrimazole and Triarylmethane-34 inhibit proliferation of cervical cancer and intrahepatic cholangiocarcinoma cells, respectively [67,68]. Piperine antagonizes IK_Ca_ channels, triggering dose-dependent G0/G1 phase arrest, proliferation suppression, and apoptosis in prostate cancer cells [69]. Blocking K_Ca_3.1 with TRAM-34 or Senicapoc slows glioma cell growth [263]. Similarly, Miconazole targeting SK_Ca_ inhibits melanoma cell growth in a dose-dependent manner [70].

The TREK-1 (K_2P_2.1) activator BL1249 inhibits proliferation and migration of PDAC cells [71]. For K_ir_ channels, Withaferin A suppresses breast cancer cell proliferation by inhibiting TASK-3 (K_2P_9.1) channel function [72]. The ATP-sensitive potassium channel (K_ATP_) is a Kir subfamily member modulated by intracellular ATP. Minoxidil triggers mitochondrial disruption and extensive DNA damage in ovarian cancer by activating K_ir_6.2 [73], and Glibenclamide acts as a blocker of K_ATP_ channels decreases proliferation and migration in endometrial adenocarcinoma cells [74].

Pharmacological modulation of K^+^ channels can enhance the efficacy of conventional anticancer agents by synergistically inducing cell death and suppressing therapy resistance. Memantine synergistically enhances cytarabine-induced cell death in acute leukemia models via K_V_1.3 inhibition [47]. Inhibition of mitochondrial K_V_1.3 channels using PCARBTP and PAPTP potently suppress cancer cell viability in vitro and synergize with Gemcitabine and Abraxane to amplify antitumor efficacy in vivo [46]. Combining BK_Ca_ blocker Penitrem A with HER-targeted agents induces synthetic lethality in breast cancer by reducing EGFR, HER2, and AKT/STAT3 activation [63]. The selective K_Ca_1.1 blocker paxilline reverses multidrug resistance in 3D-cultured sarcoma and prostate cancer spheroid models [264,265].

K^+^ channels are also emerging as promising immunotherapeutic targets. Blockade of K_ir_2.1 with ML133 has been shown to repolarize TAMs toward an antitumor phenotype, thereby suppressing tumor growth in both murine and PDX models [266]. K_Ca_3.1 (KCNN4) has been identified as a robust prognostic biomarker and predictor of immunotherapy response across multiple cancer types [267]. Mechanistically, K_Ca_3.1 activators suppress IL-10 transcription in T-cell lymphoma cells and attenuate IL-10-mediated immune evasion by reducing TAM-derived IL-10 production [268,269]. Preclinical studies further demonstrate that pharmacological blockade of K_Ca_3.1 with TRAM-34 enhances the cytotoxic activity of CAR T cells against target tumor cells [270].

### 3.4. Cl^−^ Channel Inhibitors

Recent advances in elucidating the roles of Cl^−^ channels across malignancies have catalyzed the evaluation of these channels as druggable targets for precision oncology. Tamoxifen and 5-Nitro-2-[3-phenylpropylamino]benzoic acid (NPPB), non-specific Cl^−^ channel inhibitors, suppress ClC-3 currents and cancer cell proliferation. However, their concurrent inhibition of channels such as TMEM16A/ANO1 precludes clinical utility as selective ClC-3 blockers [75,271]. Similarly, while 4,4′-diisothiocyanatostilbene-2,2•-disulfonic acid disodium salt hydrate (DIDS) enhances hyperthermia-mediated tumor suppression via Cl^−^ channel inhibition, its broad-spectrum activity raises concerns regarding off-target effects, necessitating further validation of its therapeutic applicability in oncology [272]. Chlorotoxin (ClTx), an animal venom-derived peptide initially characterized as a Cl^−^ channel blocker, specifically interacts with MMP-2 and exerts anti-invasive effect in glioma [76,273]. Qin et al. further demonstrated that ClTx-modified liposomes activate the ClC-3 channel via MMP-2 interaction, subsequently suppressing Cl^−^ currents and cell migration in gliomas [274]. Although ClC-3-specific antibodies have been proven to inhibit Cl^−^ currents [275], their antitumor efficacy requires further investigation. Conversely, ClC-3 activators like bufadienolides exert antitumor activity by activating ClC-3 channel, leading to suppression of the PI3K/Akt/mTOR signaling axis [276].

The ANO1 (TMEM16A) channel is inhibited by CaCCinh-A01, which impedes breast cancer progression [77]. Natural compounds such as schisandrathera D and vitekwangin B reduce cell viability and induce apoptosis in cancers, such as prostate cancer, by downregulating ANO1 protein expression [78,79,80]. TMEM16A-targeting natural products show broad antitumor potential in pulmonary malignancies [277], with matairesinoside and daidzein exhibiting potent inhibitory activity against lung cancer progression [81,82]. Phytochemicals like narirutin and allicin demonstrate synergistic effects with cisplatin, enhancing chemotherapeutic efficacy while reducing toxicity [278,279]. Significantly, zafirlukast, an FDA-approved asthma therapeutic, suppresses lung adenocarcinoma growth through targeted TMEM16A inhibition, revealing novel therapeutic applications for existing pharmacological agents [83]. Benzodiazepines exhibit context-dependent effects in oncology. The GABAA-R agonist lorazepam (LOR) enhances GABA-mediated signaling, promoting glioma proliferation and growth while shortening survival in diffuse midline glioma patient-derived models [280]. A retrospective cohort study in pancreatic cancer patients linked LOR to stimulated IL-6 secretion by cancer-associated fibroblasts (CAFs) and decreased survival [281]. Similarly, diazepam use in NSCLC patients correlates with poorer responses to chemoimmunotherapy compared to non-users [282]. Conversely, in a murine melanoma model, benzodiazepines synergized with radiotherapy and immune checkpoint inhibitors, enhancing overall antitumor efficacy [283]. This evidence indicates that benzodiazepines manifest dual regulatory roles in cancer. Subsequent studies should delineate their context-specific molecular mechanisms to refine potential clinical applications.

### 3.5. Trace Metal Ion Channel Inhibitors

Trace metal ion channels play essential roles in regulating cellular physiology by maintaining trace ion homeostasis, and their dysregulation has been implicated as a critical driver of tumor progression. Accordingly, targeting these channels has emerged as a promising therapeutic strategy. TRPM7 represents one of the most extensively studied candidates. Its unique bifunctional structure, encompassing both ion channel and kinase domains, provides multiple therapeutic entry points. Pharmacological inhibitors such as TG100-115 and waixenicin A, as well as gene-editing approaches, have demonstrated robust preclinical efficacy in suppressing tumor growth and metastasis [229,284,285,286]. Therapeutic targeting of TRPML1 requires consideration of tumor-specific signaling contexts. TRPML1 agonists, including ML-SA5 and MK6-83, selectively induce cancer cell death in breast, gastric, melanoma, and glioma models by triggering autophagic arrest, while sparing normal cells [234]. Conversely, TRPML1 inhibitors such as ML-SI1 promote ferroptosis in breast CSCs and enhance the chemosensitivity of breast cancer cells to doxorubicin [84].

### 3.6. Ion Transporter Inhibitors

Ion transporters play essential roles in maintaining ion balance and pH homeostasis, positioning them as attractive targets for pharmacological intervention. Cardiac glycosides such as ouabain and digoxin, which potently inhibit Na^+^/K^+^ ATPase, have demonstrated broad-spectrum antitumor activity. These agents suppress cancer cell proliferation, migration, and invasion, and can also induce tumor cell lysis [85,86,237,287]. Furthermore, their clinical use has been associated with reduced cancer risk and improved survival outcomes in certain patient populations [288,289].

Elevated cytoplasmic Ca^2+^ concentrations exert cytotoxic effects by activating cell death pathways. Inhibition of Ca^2+^-ATPases induces sustained increases in cytosolic Ca^2+^, ultimately leading to apoptosis or necrosis. For example, the selective PMCA inhibitor [Pt(O,O′-acac)(γ-acac)(DMS)] rapidly triggers apoptosis in breast cancer cells in association with cytosolic Ca^2+^ accumulation [87]. Thapsigargin (TG), a specific SERCA inhibitor, disrupts Ca^2+^ uptake into the endoplasmic reticulum, resulting in endoplasmic reticulum calcium store depletion [290]. Although TG has been extensively investigated as a chemotherapeutic candidate for prostate and other cancers, its nonselective toxicity has limited clinical translation. To improve tumor specificity, the prodrug G202 (Mipsagargin) was developed by conjugating a TG analogue to a peptide targeting prostate-specific membrane antigen (PSMA). This design restricts uptake to PSMA-expressing cells, thereby conferring tumor selectivity [291]. G202 has shown potent antitumor activity in models of prostate, breast, and bladder cancer with minimal host toxicity. A phase I clinical trial in patients with advanced solid tumors demonstrated its acceptable tolerability and favorable pharmacokinetic profile [102], while subsequent phase II studies suggested promising efficacy in hepatocellular carcinoma [103].

Research on NCX in relation to cancer remains limited. The NCX inhibitor KB-R7943 has been shown to enhance cisplatin-induced cell death in therapy-resistant ovarian carcinoma cells, without affecting sensitive counterparts, and to significantly increase radiation-induced apoptosis in medulloblastoma cells [88,89].

NHE also represent attractive therapeutic targets, with inhibitors exerting antitumor effects primarily through disruption of pH homeostasis. Representative agents include the non-specific inhibitor amiloride and the more selective derivative cariporide. Amiloride sensitizes prostate cancer cells to the tyrosine kinase inhibitor lapatinib by altering ERBB3 subcellular localization [90], and has also demonstrated antimyeloma activity [91]. Cariporide significantly enhances doxorubicin sensitivity in breast cancer cells both in vitro and in vivo [92]. Similarly, the Na^+^/K^+^/2Cl^−^ cotransporter 1 (NKCC1) has emerged as a therapeutic target. The NKCC1-specific inhibitor bumetanide exerts anti-angiogenic effects in preclinical colon cancer models and significantly suppresses the migration of metastatic glioma cells [93,94].

## 4. Limitations and Challenges

Ion channels represent a promising and rapidly expanding class of anticancer drug targets. Nevertheless, their therapeutic exploitation is still constrained by several important limitations and challenges.

First, achieving adequate drug selectivity and specificity remains a major obstacle. Ion channels are ubiquitously expressed throughout the human body, particularly in excitable tissues such as the heart and nervous system, where they mediate essential physiological functions. Nonselective inhibition therefore carries a substantial risk of off-target toxicity, including arrhythmia, hypotension, and neurotoxicity. Although efforts have increasingly focused on developing subtype-selective modulators, the high degree of structural conservation within ion channel families makes it difficult to selectively target tumor-associated subtypes without affecting their physiological counterparts.

Second, limitations inherent to current research models substantially hinder progress. Conventional in vitro systems, such as two-dimensional cell cultures, fail to recapitulate the complexity of the tumor microenvironment, including factors such as extracellular pH, hypoxia, and cell–cell interactions, all of which profoundly influence ion channel function. Consequently, compounds that appear effective in simplified models often fail in vivo. Furthermore, the absence of robust preclinical models that faithfully reproduce human ion channel characteristics and tumor–host interactions limits the predictive value of efficacy and toxicity assessments. Even in vivo, tumor regulation frequently involves multiple cooperating ion channels; thus, mono-channel inhibition may be offset by compensatory mechanisms. Adding to this complexity, certain ion channels display divergent, or even opposing, functions depending on tumor type, stage, or microenvironmental context. Spatiotemporal heterogeneity in ion concentrations (e.g., H^+^ and Ca^2+^), oxidative stress, and extracellular matrix composition within the TME further complicates drug response.

Third, the path toward clinical translation presents additional difficulties. Most ion channel-targeting drugs currently available are broad-spectrum, with limited capacity to distinguish between malignant and normal cells. This lack of specificity has led to the termination of many candidates in early-phase development due to unacceptable toxicity. Mechanisms of tumor resistance to ion channel inhibition—whether genetic, epigenetic, or adaptive—remain poorly understood, while the absence of reliable biomarkers impedes patient stratification and precision therapy. Moreover, the potential synergistic effects between ion channel inhibitors and existing treatment modalities (chemotherapy, targeted therapy, and immunotherapy) remain inadequately explored, and evidence supporting rational combination strategies is limited. Although several ion channel inhibitors have advanced to early-phase clinical trials, most studies are small, single-arm investigations with limited controls, thereby providing low-quality evidence. Finally, critical barriers related to drug distribution, metabolism, and tumor-specific delivery continue to restrict therapeutic translation.

## 5. Conclusions and Future Perspectives

Malignant tumors frequently exhibit dysregulated expression and functional alterations of ion channels. These aberrations directly contribute to oncogenic processes, including proliferation, migration, invasion, apoptosis, and therapeutic resistance, by modulating membrane potential, intracellular ion homeostasis, and downstream signaling cascades. Moreover, ion channels play critical roles in regulating immune cell activation and cytotoxic function, underscoring their importance in shaping antitumor immunity and mediating tumor–immune cell interactions. Collectively, these insights provide a strong scientific rationale for targeting ion channels as a therapeutic strategy in oncology.

Given their central role in tumor initiation and progression, ion channels have emerged as highly attractive candidates for anticancer drug development. Current strategies encompass several complementary approaches: (i) repurposing existing ion channel modulators (such as calcium channel blockers, local anesthetics, and selected natural compounds) for their antitumor properties; (ii) designing subtype-selective agents, including monoclonal antibodies and advanced drug delivery systems, to enhance specificity; and (iii) exploring rational combination therapies that integrate ion channel inhibitors with chemotherapy, targeted agents, or immunotherapies to achieve synergistic efficacy. These approaches collectively hold considerable promise for advancing precision oncology.

Addressing the current challenges in this field will require multidisciplinary collaboration and innovative methodologies. Structure-based drug design, informed by high-resolution datasets from resources such as UniProt and RCSB-PDB, will be critical for developing selective inhibitors against specific ion channel isoforms. Comparative structural analyses of conserved versus divergent domains, including pore regions, voltage-sensing modules, and ligand-binding sites, may help to discriminate tumor-associated subtypes and reduce off-target toxicity. Improved preclinical models, including tumor organoids and three-dimensional co-culture systems, can better recapitulate the tumor microenvironment and ion channel dynamics, thereby improving translational predictability. Additionally, artificial intelligence-driven tools offer new opportunities to integrate structural and functional data, predict binding affinities, and optimize subtype selectivity, thereby accelerating the discovery of safer and more effective ligands.

Future research should prioritize elucidating the structural determinants of ion channel function in cancer, developing subtype-specific modulators, and designing advanced delivery platforms to achieve tumor-selective targeting. By strengthening the interface between basic mechanistic research and clinical translation, ion channels may evolve from potential drug targets into clinically accessible therapeutic tools, ultimately expanding the landscape of precision oncology and providing novel treatment options for cancer patients.

## Figures and Tables

**Table 1:** Ion channels as pharmacological targets in cancer.

Compounds	Target	Cancer Type	Biological Effect	Molecular Mechanism	Ref.
Lidocaine	Na_V_1.5	Ovarian	EMT ↓, Sensitivity to cisplatin ↑	FAK/Paxillin pathway ↓	[18]
Lidocaine	Na_V_1.5	Glioblastoma	Migration ↓, Sensitivity to TMZ ↑	HGF/MET pathway ↓	[19]
JZTX-14	Na_V_1.5	Breast	Metastasis ↓	-	[20]
Phenytoin	Na_V_1.5	Breast	Migration ↓, Invasion ↓	-	[21]
Ropivacaine	VGSCs	Hepatocellular carcinoma	Apoptosis ↑	IL-6/STAT3 pathway ↓	[22]
JZTX-14	Na_V_1.5	Breast	Migration ↓, Invasion ↓	E-cadherin ↑, N-cadherin ↓, vimentin ↓, MMP2 ↓	[23]
Veratridine	Na_V_1.5	Colorectal	Sensitivity to 5-Fu ↑	Ca^2+^/calmodulin-dependent Ras signaling ↑	[24]
HNTX-III	Na_V_1.7	Prostate	Invasion ↓	Rho signaling pathway ↓	[25]
NESO-pAb	nNa_V_1.5	Breast	Invasion ↓	-	[24]
Bepridil	VGCCs	Ovarian	Cell viability ↓, EMT ↓	-	[26]
Bepridil	VGCCs	Chronic lymphocytic leukemia	Apoptosis ↑	NOTCH1 pathway ↓	[27]
Diltiazem	VGCCs	Breast	EMT ↓	GDF-15 ↑	[28]
KTt-45	VGCCs	Cervical	Apoptosis ↑	Caspase-3 and caspase-9 activation	[29]
BI-749327	TRPC6	Breast	Stemness ↓	Myc ↑	[30]
Cannabinoids	TRP channel	Hepatocellular carcinoma	Autophagy ↑	AMPK activation	[31]
Cannabinoids	TRP channel	Lung	Invasion ↓	ICAM-1 ↑, TIMP-1 ↑	[32]
mAb82	TRPV6	Prostate	Apoptosis ↑	-	[33]
Lacidipine	Ca_V_1.2/1.3	Breast	Antitumor immunity ↑	IDO1 ↓	[34]
Lercanidipine/amlodipine	L-type Ca^2+^ channel	Gastric	Sensitize to doxorubicin ↑	YY1/ERK/TGF-β ↓	[35]
Mibefradil	Ca_V_3.2	Ovarian	Apoptosis ↑	p-Akt ↓, FOXM1 ↓, survivin ↓	[36]
Mibefradil	Ca_V_3.2	Glioblastoma	Apoptosis ↑	mTORC2/Akt pathway ↓	[37]
SKF96365	ORAI1	Hepatocarcinoma	Autophagy ↑, Chemosensitivity to 5-Fu ↑	PI3K/AKT/mTOR pathway ↓	[38]
1B50-1	α2δ1	Small-cell lung cancer	Chemosensitivity ↑	ERK ↓	[39]
AMG9810	TRPV1	Cervical	Sensitivity to cisplatin ↑	EGFR/AKT pathway ↓	[40]
Compound D9	TRPM2	Lung	Therapeutic efficacy of osimertinib ↑	EGFR pathway ↓	[41]
Nifedipine	VGCCs	Colorectal	Proliferation ↓, Metastasis ↓, Immune escape	PD-1/PD-L1 ↓	[42]
FS48	K_V_1.1/K_V_1.3	Lung	Migration ↓, Invasion ↓	MMP-9 ↓, TIMP-1 ↑	[43]
KAaH1	K_V_1.1/K_V_1.3	Glioblastoma	Adhesion ↓, Migration ↓	-	[44]
PAP-1/PAPTP/PCARBTP	K_V_1.3	Melanoma	ROS-induced apoptosis ↑, Sensitivity to cisplatin ↑	-	[45]
PAPTP/PCARBTP	K_V_1.3	Pancreas ductal adenocarcinoma	Cell viability ↓	p38 phosphorylation ↑	[46]
Memantine	K_V_1.3	Acute lymphoblastic leukemia	Apoptosis ↑	AKT and ERK1/2 signaling ↓	[47]
Genistein	K_V_1.3	Breast	Proliferation ↓	EROD and ODC activity ↓	[48]
Genistein	K_V_1.3	Colon	Apoptosis ↑	Bax ↑, p21WAF1 ↑	[49]
Chloroquine	K_V_10.1	Breast	Migration ↓	-	[50]
Astemizole	K_V_10.1	Cervical	Autophagy ↑	-	[51]
Procyanidin B1	K_V_10.1	Liver	Proliferation ↓, Migration ↓	-	[52]
Tetrandrine	K_V_10.1	Cervical	Proliferation ↓	-	[53]
Nutlin-3	K_V_10.1	Cervical	Cell viability ↓, Migration ↓, Invasion ↓	PI3K/AKT pathway ↓	[54]
Celastrol	K_V_11.1	Prostate	Apoptosis ↑	-	[55]
Berberine	K_V_11.1	Ovarian	Proliferation ↓, Migration ↓, Invasion ↓	-	[56]
Resveratrol	K_V_11.1	Colon	Proliferation ↓, Apoptosis ↑	-	[57]
Clarithromycin	K_V_11.1	Colorectal	Apoptosis ↑	PI3K/AKT pathway ↓	[58]
Paxilline	BK_Ca_	Glioblastoma	Migration ↓, Sensitivity to cisplatin ↑	-	[59]
Iberiotoxin	BK_Ca_	Neuroblastoma	Proliferation ↓	AKT1pser473 dephosphorylation ↑	[60]
Iberiotoxin	BK_Ca_	Endometrial	Proliferation ↓, Migration ↓	-	[61]
Iberiotoxin	BK_Ca_	Hepatocellular carcinoma	Migration ↓	E-cadherin ↑, Vimentin ↓	[62]
Penitrem A	BK_Ca_	Breast	Cell-cycle arrest	p-AKT ↓, p-STAT3 ↓	[63]
Trimebutine maleate	VGCC/BK_Ca_	Ovarian	Stemness ↓	Wnt/β-catenin pathway ↓	[64]
Temozolomide	IK_Ca_	Glioma	-	-	[65]
Vigabatrin	IK_Ca_	Glioma	-	-	[66]
Clotrimazole	IK_Ca_	Cervical	Proliferation ↓	-	[67]
Triarylmethane-34	K_Ca_3.1	Intrahepatic cholangiocarcinoma	Proliferation ↓, Invasion ↓	NF-κB activation ↓	[68]
Piperine	IK_Ca_	Prostate	Cell-cycle arrest, Proliferation ↓, Apoptosis ↑	-	[69]
Miconazole	SK_Ca_	Melanoma	Proliferation ↓	-	[70]
BL1249	K_2P_2.1	Pancreatic	Proliferation ↓, Migration ↓	-	[71]
Withaferin A	TASK-3	Breast	Proliferation ↓	-	[72]
Minoxidil	K_ir_6.2	Ovarian	Tumor growth ↓	-	[73]
Glibenclamide	K_ATP_	Endometrial adenocarcinoma	Proliferation ↓, Migration ↓	-	[74]
NPPB	ClC-3	Nasopharyngeal carcinoma	-	-	[75]
Chlorotoxin	ClC-3	Glioma	Invasion ↓	MMP-2 ↓	[76]
CaCCinh-A01	ANO1	Breast	Cell viability ↓	EGFR and CAMK signaling ↓	[77]
Schisandrathera D	ANO1	Prostate	Apoptosis ↑	Caspase-3 ↑	[78]
Vitekwangin B	ANO1	Prostate	Apoptosis ↑	-	[79]
Cis-resveratrol	ANO1	Prostate	Proliferation ↓, Migration ↓	Caspase-3 ↑	[80]
Matairesinoside	ANO1	Lung	Migration ↓, Invasion ↓, Apoptosis ↑	-	[81]
Daidzein	ANO1	Lung	Cell viability ↓, Migration ↓, Cell-cycle arrest ↑	-	[82]
Zafirlukast	ANO1	Lung	Proliferation ↓, Migration ↓	-	[83]
ML-SI1	TRPML1	Breast	Ferroptosis ↑, Stemness ↓, Sensitivity to doxorubicin ↑	-	[84]
Ouabain	Na^+^/K^+^ ATPase	Breast	Cell lysis ↑	-	[85]
Ouabain	Na^+^/K^+^ ATPase	Breast	Proliferation ↑, Motility ↑, Invasion ↑	p-Rac/cdc42 ↓, p-ERK1/2 ↓	[86]
DMS	PMCA	Breast	Apoptosis ↑	-	[87]
KB-R7943	NCX	Ovarian	Sensitivity to cisplatin ↑	-	[88]
KB-R7943	NCX	Medulloblastoma	Apoptosis ↑	-	[89]
Amiloride	NHE	Prostate	Sensitivity to lapatinib ↑	-	[90]
Amiloride	NHE	Multiple myeloma	Apoptosis ↑	p53 signaling ↑	[91]
Cariporide	NHE	Breast	Apoptosis ↑	MDR1 ↑, Cleaved caspase 3/9 ↑	[92]
Bumetanide	NKCC1	Colon	Vascularity ↓	CD31 ↓, VEGF ↓	[93]
Bumetanide	NKCC1	Glioma	Migration ↓, Invasion ↓	-	[94]

Arrows indicate the following: ↑, upregulation/activation; ↓, downregulation/inhibition.

**Table 2:** Clinical trials of anticancer drugs targeting ion channels.

ClinicalTrials.gov ID.	Phase	Agents	Status	Cancer Types	End Points	Ref.
NCT01916317	III	Lidocaine	Completed	Breast	DFS and OS	[95]
NCT02786329	I	Lidocaine	Completed	Colorectal	Recurrence rate	[15]
NCT04761614	I	Riluzole	Completed	Colorectal	Safety profile	[96]
NCT01480050	I	Mibefradil	Completed	Gliomas	Safety and the maximum tolerated dose	[16]
NCT01578564	I	SOR-C13	Completed	Advanced solid tumors	Safety/tolerability and pharmacokinetics	[97]
NCT05629702	II	Cannabinoids	Recruiting	Recurrent glioblastoma	OS	[98]
NCT02587819	I	nfP2X7-targeted antibodies	Completed	Basal cell carcinoma	Safety, tolerability and pharmacokinetics	[99]
NCT01107522	IB	Carboxyamidotriazole	Completed	Recurrent and newly diagnosed glioblastoma	Safety profile	[100]
CTR20160395	III	Carboxyamidotriazole	Completed	Advanced non-small-cell lung cancer	PFS	[101]
NCT01056029	I	Mipsagargin	Completed	Advanced solid tumors	Safety, the maximum tolerated dose and pharmacokinetics	[102]
NCT01777594	II	Mipsagargin	Completed	Progressive advanced hepatocellular carcinoma	Response rate, PFS, OS	[103]
-	I	Carboxyamidotriazole	Completed	Refractory solid tumors	Pharmacokinetic analysis	[17]
-	II	Carboxyamidotriazole	Completed	Relapsed epithelial ovarian cancer	Safety, tolerability and pharmacokinetics	[104]

## Data Availability

No new data were created or analyzed in this study. Data sharing is not applicable.

## Abbreviations

The following abbreviations are used in this manuscript:

ASICs	Acid-sensing ion channels
BK_Ca_	Large-conductance calcium-activated potassium channel
CaCCs	Calcium-activated chloride channels
CAF	Cancer-associated fibroblast
CCBs	Calcium channel blockers
CF	Cystic fibrosis
CFTR	Cystic fibrosis transmembrane conductance regulator
CSC	Cancer stem cell
ECM	Extracellular matrix
EMT	Epithelial-mesenchymal transition
ENaCs	Epithelial Na^+^ channels
GABAA-Rs	Gamma-aminobutyric acid type A receptors
GlyRs	Glycine receptors
HCC	Hepatocellular carcinoma
HNSCC	Head and neck squamous cell carcinoma
IK_Ca_	Intermediate-conductance calcium-activated potassium channel
K_2P_	Two-pore domain potassium channels
K_Ca_	Calcium-activated potassium channels
K_ir_	Inwardly rectifying potassium channels
KV	Voltage-gated potassium channels
LGCCs	Ligand-gated calcium channels
LGClCs	Ligand-gated chloride channels
NPC	Nasopharyngeal carcinoma
NSCLC	Non-small-cell lung cancer
OSCC	Oral squamous cell carcinoma
PDAC	Pancreatic ductal adenocarcinoma
SCLC	Small-cell lung cancer
SK_Ca_	Small-conductance calcium-activated potassium channel
SOCCs	Store-operated calcium channels
SOCE	Store-operated calcium entry
TAMs	Tumor-associated macrophages
TME	Tumor microenvironment
TMs	Transmembrane domains
TMZ	Temozolomide
TNBC	Triple-negative breast cancer
TRP	Transient receptor potential
TTX	Tetrodotoxin
VGCCs	Voltage-gated calcium channels
VGSC	Voltage-gated sodium channel
VRACs	Volume-regulated anion channels
